# A Sequential Optimization Calibration Algorithm for Near-Field Source Localization

**DOI:** 10.3390/s17061405

**Published:** 2017-06-15

**Authors:** Jingjing Li, Xianxiang Yu, Guolong Cui

**Affiliations:** 1 Kexin College, Hebei University of Engineering, Handan 056038, China; woaichoupi@126.com; 2 School of Electronic Engineering, University of Electronic Science and Technology of China, Chengdu 611731, China; cuiguolong@uestc.edu.cn

**Keywords:** near-field source location problem, sensor gain and phase errors, a sequential optimization calibration method

## Abstract

This paper considers the near-field source location problem for a nonuniform linear array (non-ULA) in the presence of sensor gain and phase errors. A sequential optimization calibration method is proposed to simultaneously estimate the gain and phase errors as well as the locations of calibration sources involving the ranges and the azimuths by exploiting some imprecise a-priori knowledge of calibration sources. At each iteration of the proposed method, the source locations, and the gain and phase errors are obtained iteratively. Finally, at the analysis stage, we evaluate the effectiveness of the proposed technique using some numerical simulations. Results show that the proposed algorithm shares the capability to jointly estimate the source locations and the errors.

## 1. Introduction

Source localization with sensor arrays has broad applications in aerospace, navigation and wireless acoustic sensor network societies [1,2,3,4,5,6]. Many effective algorithms for source locations, such as the maximum-likelihood (ML) [7], the multiple signal classification (MUSIC) [8] and the minimum-variance distortionless response (MVDR) algorithms [9], have been developed over the years. Recently, some source location algorithms based on cumulative sum and steered-response power [4,5,10,11,12] are also presented. It is worth pointing out that most of these algorithms suppose the array manifold is perfectly available. However, in practice, the array manifold is often affected by unknown array characteristics such as the sensor gain and phase errors as well as the unknown mutual coupling [13,14,15], thus resulting in the performance deterioration of these algorithms. For example, in [14], the precision of the direction-of-arrival (DOA) estimation would decrease due to the existence of the unknown mutual coupling. In [15], the accuracy of measuring acoustic intensity would degrade in the presence of the sensor gain and phase errors.

Some calibration algorithms for the gain and phase errors have been investigated. Specifically, in [16,17], the eigendecomposition method was proposed to derive the DOAs with unknown gain and phase errors. In [18], using the null characteristic of the MUSIC spectrum, a calibration technique was proposed for the sensor gain and phase uncertainties as well as location errors. In [19], the maximum a posteriori (MAP) method was presented to estimate the DOAs and the perturbations simultaneously. In [20], an eigenstructure method with the aim of simultaneously estimating the DOA and gain-phase errors without joint iteration, was proposed. In [21], based on different data models, two new estimation algorithms were presented for uniform linear array (ULA) to estimate sensor gain and phase errors. In [22], exploiting the subspace principle, the estimations of sensor gain and phase errors were addressed in subarrays-based linear sparse arrays. In [23], a new method based on the eigendecomposition of the Hadamard product of the covariance matrix and its conjugate was investigated requiring no a priori knowledge of calibration sources. In [24], the authors presented an estimation of signal parameters via rotational invariance technique (ESPRIT)-like method that can simultaneously estimate DOA, as well as the gain and phase errors in the uncalibrated portion of the ULA in close form.

Summarizing, the aforementioned works focus on the far-field scenario, where the sources are located far enough from the sensor arrays and only the DOAs are of interest. In the case of the near-field scenario, the steering vector involves the knowledge of both the azimuths and the ranges. Some works, such as [25,26,27,28,29,30,31], have been presented to obtain the ranges and DOAs of the sources but with perfect knowledge of the array manifold. However, in the presence of the sensor gain and phase errors, the estimation performance would be degraded significantly. There is very little work with investigating the near-field source location problem for a nonuniform linear array (non-ULA) in the presence of sensor gain and phase errors. In [32], the passive localization of near-field sources with partly calibrated subarray-based arrays was studied. However, the proposed method cannot apply in arbitrary arrays. We can employ the traditional active-calibration methods to estimate the gain and phase errors no matter if they are far-field targets or near-field targets requiring the exact information (azimuth or range) of the calibration source. However, from a practical point of view, the exact information cannot be available.

In this paper, we consider the near-field source location problem for a non-ULA in the presence of sensor gain and phase errors. We present a sequential optimization calibration method to simultaneously estimate the error parameters and the locations of calibration sources under minimum variance estimation criterion based on some imprecise a priori knowledge of calibration sources. Specifically, we first obtain the estimates of source azimuths, the gain and phase errors using given ranges. Second, the source ranges and the error parameters are derived according to the known azimuths. Finally, we continue the iterative procedure until convergence. Simulation results highlight that the proposed algorithm shares the capability of the joint estimates of the source locations and the error parameters. In particular, it is worth pointing out that the proposed method exploits the imprecise knowledge of calibration sources to estimate gain and phase errors. As a consequence, it is more effective than the active-calibration methods owing to combing the self-calibration and active-calibration techniques.

The rest of the paper is organized as follows. In Section 2, we formulate the signal model. Section 3 describes a self-calibrating method for near-field sources with unknown gain and phase errors. In Section 4, we provide some simulations to illustrate the effectiveness of the proposed method. Finally, in Section 5, some conclusions are derived.

## 2. Signal Model

**Figure 1:**
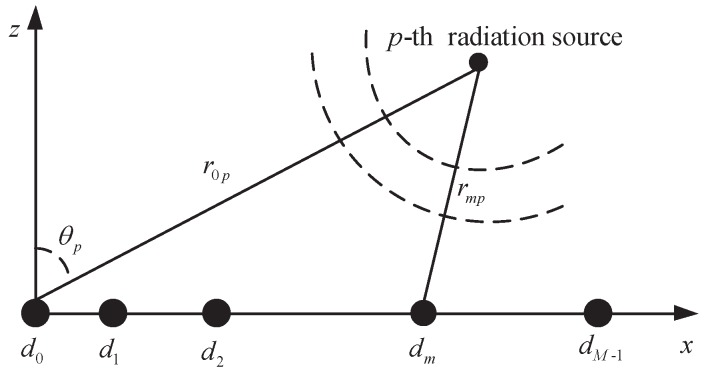
Diagram showing narrow-band non-ULA architecture.

Assume that a non-ULA contains *M* sensors, which are placed along the *x*-axis at $d_{0},d_{1},\cdots ,d_{M-1}\left(d_{0}=0\right)$ with unequal spacing, respectively, as shown in Figure 1. There are *P* uncorrelated narrow-band near-field sources impinging on the SLA with azimuth and range pairs $\left(\theta_{p},r_{0p}\right)$, p=1,2,…,P, where $\theta_{p}$ is the azimuth of the *p*-th source deviating normal direction of array reference point and $r_{0p}$ is the distance between the *p*-th source and array reference point. Let $s_{p}\left(n\right)$, n=1,2,…,L, be the source waveforms. Similar to [33], the received signal vector of the non-ULA at the *n*th snapshot can then be expressed as,

(1) $$\begin{array}{cc} x(n) & =\sum_{p=1}^{P}\Gamma a\left(\theta_{p},r_{0p}\right)s_{p}\left(n\right)+v\left(n\right)\\ & =\Gamma As(n)+v(n)\;\;\;\;\;n=1,2,\cdots ,L, \end{array}$$

where

- $x\left(n\right)={\left[x_{0}\left(n\right),x_{1}\left(n\right),\cdots ,x_{M-1}\left(n\right)\right]}^{T}$ is the measurement signal vector;
- $v\left(n\right)={\left[v_{0}\left(n\right),v_{1}\left(n\right),\cdots ,v_{M-1}\left(n\right)\right]}^{T}$ is an independent and identically distributed complex circular zero-mean Gaussian random vector with covariance matrix $\sigma^{2}I_{M}$, while $I_{M}$ is the *M*-dimensional identity matrix;
- $A=[a\left(\theta_{1},r_{01}\right),a\left(\theta_{2},r_{02}\right),\cdots ,a\left(\theta_{P},r_{0P}\right)]$ is the nominal M×P steering matrix, the *p*-th column is
  (2) $$\begin{array}{c} a\left(\theta_{p},r_{0p}\right)=\left[1,\frac{r_{0p}}{r_{1p}}e^{-j\frac{2\pi \Delta r_{1p}}{\lambda_{p}}},\cdots ,\right.\\ {\left.\frac{r_{0p}}{r_{(M-1)p}}e^{-j\frac{2\pi \Delta r_{(M-1)p}}{\lambda_{p}}}\right]}^{T}, \end{array}$$
  where $\lambda_{p}$ is the wavelength of the *p*th source and $r_{mp}$ denotes distance of the *p*th signal source to *m*th sensor, p=1,2,⋯,P, m=0,1,⋯,M-1. $\Delta r_{mp}$ is the relative distance between $r_{0p}$ and $r_{mp}$, which can be derived by geometrical relationship,
  (3) $$\begin{array}{cc} \Delta r_{mp} & =r_{0p}-r_{mp}\\ & =r_{0p}-\sqrt{{\left(r_{0p}\sin\theta_{p}-d_{m}\right)}^{2}+{\left(r_{0p}\cos\theta_{p}\right)}^{2}}. \end{array}$$
  To simplify the notation, we write $r_{0p}$ into $r_{p}$.
- $\Gamma =\mathrm{diag}\{\rho_{0}e^{j\varphi_{0}},\rho_{1}e^{j\varphi_{1}},\cdots ,\rho_{M-1}e^{j\varphi_{M-1}}\}$ is the error matrix of the array gain and phase, where parameters $\rho_{m}$ and $\varphi_{m}$ are the gain and the phase errors associated with the *m*-th sensor, respectively.

## 3. Near-Field Calibration Method

Unlike [17], which investigated the calibration algorithm for the far-field sources where only the DOAs are of interest, this paper focuses on the case of the near-field sources involving these considerations of range and azimuth. To this end, we present a sequential optimization calibration technique to estimate the gain and phase errors, and the locations of radiating sources, simultaneously. Specifically, fixed the ranges of radiating sources, we first estimate the azimuths, the gain and phase errors, and then obtain ranges and the error parameters exploiting the estimated azimuths, and continue the procedure until convergence.

### 3.1. Joint Estimations of Error Matrix and Azimuthes with Known Ranges

In this subsection, we extend the method [16] to simultaneously estimate $\Gamma_{\theta }$ and ${\left\{\theta_{p}\right\}}_{p=1}^{P}$ using estimated ranges ${\left\{{\hat{r}}_{p}\right\}}_{p=1}^{P}$, where $\Gamma_{\theta }$ is the array gain and phase error matrix under known ranges and ${\hat{r}}_{p}$ denotes the estimated value of $r_{p}$. Precisely, given a $\Gamma_{\theta }$, we look for *P* peak values corresponding to ${\left\{\theta_{p}\right\}}_{p=1}^{P}$, with respect to one-dimensional MVDR spectrum [9], given by,

(4) $$\begin{array}{c} P\left(\theta |{\hat{r}}_{p},\Gamma_{\theta }\right)=\frac{1}{a^{H}\left(\theta ,{\hat{r}}_{p}\right)\Gamma_{\theta }^{H}R_{x}^{-1}\Gamma_{\theta }a\left(\theta ,{\hat{r}}_{p}\right)},\\ p=1,2,\cdots ,P. \end{array}$$

Using the ${\left\{\theta_{p}\right\}}_{p=1}^{P}$, we estimate $\Gamma_{\theta }$ based on minimum variance estimation criterion [9] and proceed the iteration procedure until convergence. Specifically, given ${\left\{\theta_{p}\right\}}_{p=1}^{P}$, we extend the proposed self-calibration algorithm [16] and develop a cost function computed as,

(5) $$J\left(\Gamma_{\theta }\right)=\sum_{p=1}^{P}a^{H}\left(\theta_{p},{\hat{r}}_{p}\right){\Gamma_{\theta }}^{H}R_{x}^{-1}\Gamma_{\theta }a\left(\theta_{p},{\hat{r}}_{p}\right),$$

where $a(\theta_{p},{\hat{r}}_{p})$ denotes the steering manifold in Label (2) with known range ${\hat{r}}_{p}$ and $R_{x}$ is covariance matrix computed as,

(6) $$R_{x}=E\left[xx^{H}\right]=\Gamma_{\theta }AR_{s}A^{H}{\Gamma_{\theta }}^{H}+\sigma^{2}I_{M},$$

while $R_{s}=E\left[ss^{H}\right]$, E[·] and ${\left(\cdot \right)}^{H}$ represent the expectation and the Hermitian transpose operation, respectively. Note that the number *P* of signal source can be estimated based on Schwartz and Rissanen (MDL) criteria [34] and $R_{x}$ can be estimated by the sample covariance matrix (i.e., $R_{x}=\sum_{n=1}^{N}x\left(n\right)x^{H}\left(n\right)$, where *N* is the number of snapshots).

Further, let

(7) $$\Gamma_{\theta }a\left(\theta_{p},{\hat{r}}_{p}\right)=\tilde{a}\left(\theta_{p},{\hat{r}}_{p}\right)\delta ,$$

in which

(8) $$\Gamma_{\theta }=\mathrm{diag}\left(\delta \right),$$

and $\tilde{a}\left(\theta_{p},r_{p}\right)$ is a diagonal matrix, given by,

(9) $$\tilde{a}\left(\theta_{p},{\hat{r}}_{p}\right)=\mathrm{diag}\left\{a\left(\theta_{p},{\hat{r}}_{p}\right)\right\},$$

while diag(·) denotes diagonal matrix formed by the entries of the vector.

Submitting Label (7) into Label (5), we have

(10) $$J\left(\Gamma_{\theta }\right)=\delta^{H}\left\{\sum_{p=1}^{P}\tilde{a}{\left(\theta_{p},{\hat{r}}_{p}\right)}^{H}R_{x}^{-1}\tilde{a}\left(\theta_{p},{\hat{r}}_{p}\right)\right\}\delta .$$

Hence, assuming that $\rho_{0}=1$,$\varphi_{0}=0$, the optimization problem $P_{1}$ accounting for the constraint $\delta^{H}w=1$ where $w={\left[1,0,\ldots ,0\right]}^{T}$, can be written as,

(11) $$P_{1}\left\{\begin{array}{cc} \min_{\Gamma_{\theta }} & J(\Gamma_{\theta })\\ s.t. & \delta^{H}w=1,\\ & \Gamma_{\theta }=\mathrm{diag}\left(\delta \right). \end{array}\right.$$

Employing the Lagrangian multiplier method, the optimal solution to $P_{1}$ is derived as:

(12) $$\delta =\frac{Q^{-1}w}{w^{T}Q^{-1}w},$$

where

(13) $$Q=\sum_{p=1}^{P}\tilde{a}{\left(\theta_{p},{\hat{r}}_{p}\right)}^{H}R_{x}^{-1}\tilde{a}\left(\theta_{p},{\hat{r}}_{p}\right).$$

Summarizing, the procedure of joint estimating $\Gamma_{\theta }$ and source azimuths ${\left\{\theta_{p}\right\}}_{p=1}^{P}$ is summarized in Algorithm 1:
**Algorithm 1** Algorithm for the joint estimations of $\Gamma_{\theta }$ and θ.**Input:**$\left(d_{0},d_{1},\ldots ,d_{M-1}\right)$, ${\left\{r_{p}\right\}}_{p=1}^{P}$, ***w***, *P*, $R_{x}$.**Output:**${\left\{\theta_{p}\right\}}_{p=1}^{P}$ and $\Gamma_{\theta }$.1:For k=0 and $\Gamma^{\left(0\right)}=I_{M}$;2:Find the azimuths ${\left\{\theta_{p}^{\left(0\right)}\right\}}_{p=1}^{P}$ by searching for *P* highest peak using Label (4);3:Compute $J_{0}$ by Label (10);4:k:=k+1;5:Compute $Q^{\left(k\right)}$ by Label (13);6:Compute $\delta^{\left(k\right)}$ by Label (12);7:Construct $\Gamma^{\left(k\right)}=\mathrm{diag}\left\{\delta^{\left(k\right)}\right\}$;8:Find the azimuths ${\left\{\theta_{p}^{\left(k\right)}\right\}}_{p=1}^{P}$ by searching for *P* highest peak using Label (4);9:Compute $J_{k}$ by Label (10);10:If $|J_{k}-J_{k-1}|>\kappa$, where κ is a user selected parameter to control convergence, then k=k+1, return to step 4. Otherwise, stop and output $\Gamma_{\theta }=\Gamma^{\left(k\right)}$, ${\left\{\theta_{p}\right\}}_{p=1}^{P}={\left\{\theta_{p}^{\left(k\right)}\right\}}_{p=1}^{P}$.

### 3.2. Joint Estimations of Error Matrix and Ranges with Known Azimuths

In this subsection, we focus on jointly estimating $\Gamma_{r}$ and ${\left\{r_{p}\right\}}_{p=1}^{P}$ with known azimuths ${\left\{{\hat{\theta }}_{p}\right\}}_{p=1}^{P}$, where $\Gamma_{r}$ is the array gain and phase error matrix under known azimuths and ${\hat{\theta }}_{p}$ denote the estimated value of $\theta_{p}$. Specifically, given a $\Gamma_{r}$, we first find ${\left\{r_{p}\right\}}_{p=1}^{P}$ by searching for one-dimension MVDR spectrum, given by

(14) $$\begin{array}{c} P\left(r|{\hat{\theta }}_{p},\Gamma_{r}\right)=\frac{1}{a^{H}\left({\hat{\theta }}_{p},r\right)\Gamma_{r}^{H}R_{x}^{-1}\Gamma_{r}a^{H}\left({\hat{\theta }}_{p},r\right)},\\ p=1,2,\cdots ,P. \end{array}$$

Then, according to the knowledge ${\left\{r_{p}\right\}}_{p=1}^{P}$, we employ the same above procedure to derive $\Gamma_{r}$ and continue the procedure until convergence.

Next, we focus on the derivation of $\Gamma_{r}$ with obtained ${\left\{r_{p}\right\}}_{p=1}^{P}$. Similarly, the optimal problem $P_{2}$ can be denoted as

(15) $$P_{2}\left\{\begin{array}{cc} \min_{\Gamma_{r}} & J(\Gamma_{r}),\\ s.t. & \delta^{H}w=1,\\ & \Gamma_{r}=\mathrm{diag}\left(\delta \right), \end{array}\right.$$

where

(16) $$J\left(\Gamma_{r}\right)=\delta^{H}\left\{\sum_{p=1}^{P}\tilde{a}{\left({\hat{\theta }}_{p},r_{p}\right)}^{H}R_{x}^{-1}\tilde{a}\left({\hat{\theta }}_{p},r_{p}\right)\right\}\delta ,$$

and

(17) $$\tilde{a}\left({\hat{\theta }}_{p},r_{p}\right)=\mathrm{diag}\left\{a\left({\hat{\theta }}_{p},r_{p}\right)\right\}.$$

We can derive the solution of $P_{2}$ as

(18) $$\delta =\frac{Q^{-1}w}{w^{T}Q^{-1}w},$$

where

(19) $$Q=\sum_{p=1}^{P}\tilde{a}{\left({\hat{\theta }}_{p},r_{p}\right)}^{H}R_{x}^{-1}\tilde{a}\left({\hat{\theta }}_{p},r_{p}\right).$$

Finally, Algorithm 2 summarizes the procedure of jointly estimating $\Gamma_{r}$ and source ranges ${\left\{r_{p}\right\}}_{p=1}^{P}$.
**Algorithm 2** Algorithm for the joint estimations of $\Gamma_{r}$ and $r_{p}$**Input:**$\left(d_{0},d_{1},\ldots ,d_{M-1}\right)$, ${\left\{\theta_{p}\right\}}_{p=1}^{P}$, ***w***, *P*, $R_{x}$;**Output:**${\left\{r_{p}\right\}}_{p=1}^{P}$ and $\Gamma_{r}$;1:For k=0 and $\Gamma^{\left(0\right)}=I_{M}$;2:Find the ranges ${\left\{r_{p}^{\left(0\right)}\right\}}_{p=1}^{P}$ by searching for *P* highest peak using Label (14);3:Compute $J_{0}$ by Label (16);4:k:=k+1;5:Compute $Q^{\left(k\right)}$ by Label (19);6:Compute $\delta^{\left(k\right)}$ by Label (18);7:Construct $\Gamma^{\left(k\right)}=\mathrm{diag}\left\{\delta^{\left(k\right)}\right\}$;8:Find the ranges ${\left\{r_{p}^{\left(k\right)}\right\}}_{p=1}^{P}$ by searching for *P* highest peak using (14);9:Compute $J_{k}$ by Label (16);10:If $|J_{k}-J_{k-1}|>\kappa$, then k=k+1, return to step 4. Otherwise, stop and output $\Gamma_{r}=\Gamma^{\left(k\right)}$, ${\left\{r_{p}\right\}}_{p=1}^{P}={\left\{r_{p}^{\left(k\right)}\right\}}_{p=1}^{P}$.

### 3.3. Joint Estimations of Error Matrix, Azimuths and Ranges

In this subsection, the proposed iteration procedure for jointly estimating the source range and azimuth $\left(\theta_{p},r_{p}\right)$, p=1,2,⋯,P, as well as the gain and phase error matrix Γ is summarized in Algorithm 3. It is worth mentioning that the calibration algorithm based on eigenstructure methods in [17] can also be used to calibrate the near-field sources. However, each iteration of the calibration algorithm requires to search a two-dimensional pseudo-specturm resulting in a large computational complexity. In particular, each iteration of the proposed algorithm provides a computationally efficient calibration method through converting a two-dimensional spectrum calibration problem into two one-dimensional spectrum calibration problems, which significantly decreases the computational burden.

Finally, we point out that the proposed algorithm may not converge to an optimal solution since both optimization problems (4) and (14) are not convex. The proposed iterative algorithm ensures obtaining a quality suboptimal solution as the approximate solution of optimal solution (please see Table 1).
**Algorithm 3** Algorithm for the joint estimation of Γ and ${\left\{\theta_{p}\right\}}_{p=1}^{P},{\left\{r_{p}\right\}}_{p=1}^{P}$
**Input:**$\left(d_{0},d_{1},\ldots ,d_{M-1}\right)$, ***w***, $\Gamma_{0}$, $R_{x}$;**Output:**Γ and $\left(\theta_{p},r_{p}\right)$, p=1,2,…,P;1:For m=0 and estimate the number *P* of signals;2:Find the P peaks corresponding to locations $\left(\theta_{p}^{\left(0\right)},r_{p}^{\left(0\right)}\right)$, p=1,2,…,P by searching for two-dimension MVDR spectrum $P_{c}\left(\theta ,r|\Gamma^{\left(0\right)}\right)=1/a^{H}\left(\theta ,r\right){\left(\Gamma_{0}\right)}^{H}R_{x}^{-1}\Gamma_{0}a\left(\theta ,r\right)$;3:m:=m+1;4:Estimate ${\left\{\theta_{p}^{\left(m+1\right)}\right\}}_{p=1}^{P}$ and $\Gamma_{\theta }$ by Algorithm 1 using ${\left\{r_{p}^{\left(m\right)}\right\}}_{p=1}^{P}$;5:Estimate ${\left\{r_{p}^{\left(m+1\right)}\right\}}_{p=1}^{P}$ and $\Gamma_{r}$ by Algorithm 2 using ${\left\{\theta_{p}^{\left(m+1\right)}\right\}}_{p=1}^{P}$;6:If $∥\Gamma_{\theta }-\Gamma_{r}{∥}_{F}^{2}>\kappa$, where ${∥\cdot ∥}_{F}$ denotes matrix 2-norm, back to step 3; Otherwise, stop and output $\Gamma =\Gamma_{\theta }$, $\left(r_{p},\theta_{p}\right)=\left(r_{p}^{\left(m+1\right)},\theta_{p}^{\left(m+1\right)}\right)$, p=1,2,…,P.

## 4. Numerical Results

In this section, we evaluate the performance of the proposed algorithm via numerical simulations. We suppose that the non-ULA composes of *M* = 12 isotropous elements randomly placed in the array aperture D=100 m with the working wavelength λ=0.15 m and N=1000. Additionally, we model the array gain errors and phase errors as random variables obeying uniform distribution, which are generated by [22]:

$$\rho_{m}=1+\sqrt{12}\sigma_{p}\zeta_{m},$$

$$\varphi_{m}=\sqrt{12}\sigma_{\varphi }\eta_{m},$$

where $\zeta_{m}$ and $\eta_{m}$ are independent and identically distributed random variables distributed uniformly over [-0.5,0.5], $\sigma_{p}$ and $\sigma_{\varphi }$ are the standard deviations of $\rho_{m}$ and $\varphi_{m}$, respectively. Finally, the exit condition for Algorithms 1–3 is $\kappa =10^{-4}$.

### 4.1. The Joint Estimations of the Array Gain and Phase Errors and Source Locations

In this subsection, we focus on jointly estimating the array gain and phase errors and source locations using the imprecise location knowledge of calibration sources. Specifically, without loss of generality, we consider a scenario involving three calibration sources (note that we can exploit a calibration source or two calibration sources) located at $\left(1400\;m,10^{\circ }\right),\left(1500\;m,22.5^{\circ }\right),\left(1600\;m,-15^{\circ }\right)$, respectively assuming that all signal-to noise ratio (SNRs) are 20 dB. In particular, we suppose the imprecise distances (this is reasonable due to the measurement error) of the three sources are 1385m,1485m,1615m, respectively, which are chosen as initializations of Algorithm 3. Additionally, we randomly generate $L_{1}=50$ experiments for gain and phase errors under $\sigma_{p}=0.1$ and $\sigma_{\varphi }=\pi /18$. For each experiment corresponding to a set of fixed gain and phase errors, we conduct $L_{2}=50$ Monte Carlo trials for eliminating the impact on the noise. To this end, the RMSE (Root Mean Square Error) of the gain and phase errors are defined as, respectively,

(20) $${\mathrm{RMSE}}_{1}=\sqrt{\frac{\sum_{l=1}^{L_{2}}\sum_{m=1}^{M}{\left|\rho_{m}-\rho_{m}^{\left(l\right)}\right|}^{2}}{ML_{2}}},$$

(21) $${\mathrm{RMSE}}_{2}\left(\mathrm{in}\;\deg\right)=\sqrt{\frac{\sum_{l=1}^{L_{2}}\sum_{m=1}^{M}{\left|\varphi_{m}-\varphi_{m}^{\left(l\right)}\right|}^{2}}{ML_{2}}},$$

where $\rho_{m}^{\left(l\right)}$ and $\varphi_{m}^{\left(l\right)}$, respectively, are estimation values of the gain and phase errors of *m*-th sensor for *l*-th Monte Carlo trial.

**Figure 2:**
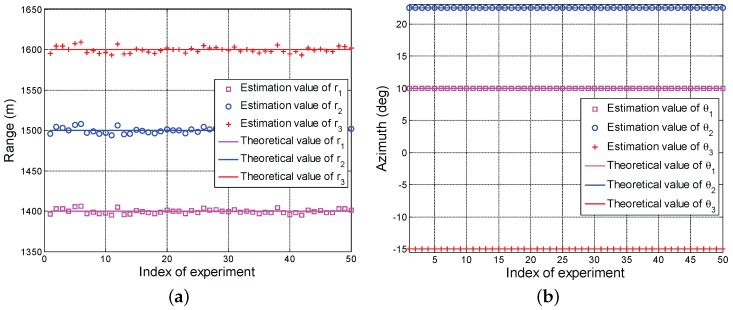
Average estimation values of the range and the azimuth of calibration source versus different experiments, (**a**) range; (**b**) azimuth.

Figure 2 depicts the average estimation values of the range and the azimuth of calibration source versus different experiments. Note that the obtained results are average over 50 Monte Carlo trials. We observe that all range estimation values of three calibration sources hover around their true ranges in Figure 2a, respectively. In particular, the maximum estimation error is about 9 m. This is reasonable since the obtained solution is suboptimal in Algorithm 3. Interestingly, in Figure 2b, all azimuth estimation values overlap perfectly with the theoretical values. These performance behaviors indicate that the proposed algorithm can accurately estimate the azimuth of calibration source but sharing a slight error in range.

**Figure 3:**
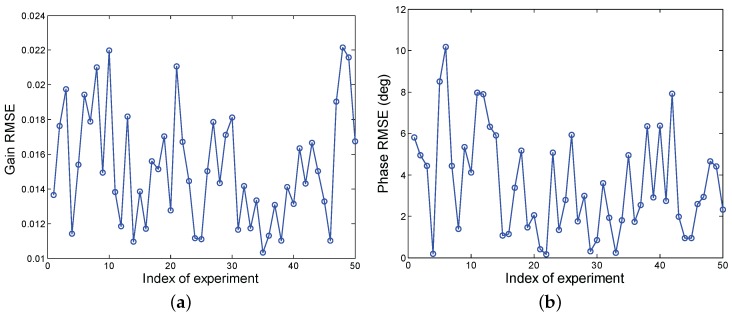
RMSEs of gain and phase errors versus different experiments, (**a**) gain error; (**b**) phase error.

Figure 3 shows RMSEs of the gain and phase errors versus different experiments. Results reveal that the different gain and phase errors would result in different estimation errors due to the existence of range estimation errors. In particular, it can be observed that the mean RMSEs of the gain and phase errors are about 0.015 and $3.5^{\circ }$, respectively. Finally, it is worth pointing out that the proposed technique shares the capability of the robustness to estimate the different gain and phase errors in correspondences of the analyzed parameters.

Next, we randomly select a set of values of the gain and phase errors among 50 experiments. In Table 1, we report the theoretical and average estimated values of the gain and phase errors for 12 sensors, where ρ and $\hat{\rho }$ are respectively theoretical and estimated values of the gain error, and φ and $\hat{\varphi }$ are, respectively, theoretical and estimated values of the phase error. Note that the estimated values for each sensor are average results of 50 Monte Carlo trials. Interestingly, it can be seen that the estimated values are close to the true values showing that the proposed technique can well estimate the gain and phase errors.

Next, we analyze the MVDR spectrum given by

(22) $$\begin{array}{c} P\left(r,\theta \right)=\frac{1}{a^{H}\left(\theta ,r\right)\Gamma^{H}R_{x}^{-1}\Gamma a\left(\theta ,r\right)}. \end{array}$$

**Figure 4:**
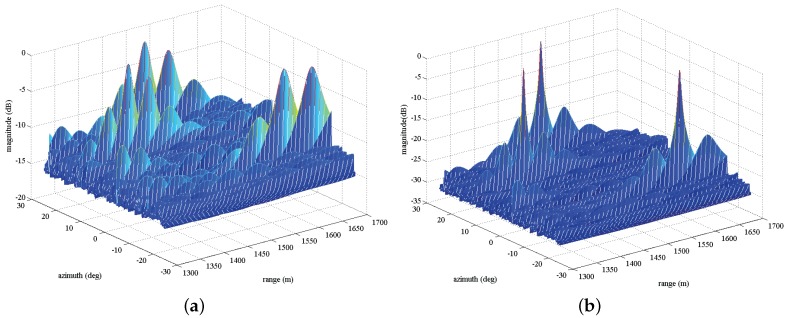
Two-dimensional MVDR spatial spectrums for three calibration sources, (**a**) before calibration; (**b**) after calibration.

In Figure 4, we plot the two-dimensional MVDR spatial spectrums for three calibration sources considering two cases of before correction (Figure 4a) and after correction (Figure 4b). It can be observed that the high sidelobe level emerges around the locations of true targets before calibration due to the nominal steering vector imperfectly matching the real one when existing the gain and phase errors. After calibration, as expected, the sidelobe levels significantly decrease and three notable peaks can be observed.

**Figure 5:**
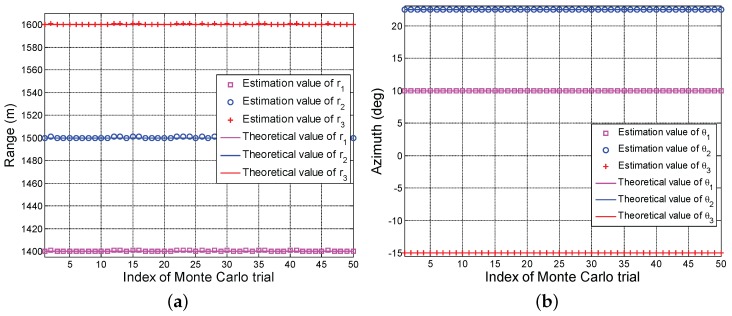
Estimation values of range and azimuth of calibration sources versus different Monte Carlo trials, (**a**) range; (**b**) azimuth.

In Figure 5, we show the estimation values of range and azimuth of calibration source for 50 Monte Carlo trials. Again, we observe that the obtained ranges show a slight oscillatory in comparison with the true ranges, whereas the estimated azimuths perfectly overlap with true values. These performance behaviors reveal that the proposed technique can better estimate the locations of calibration sources.

### 4.2. Array Gain and Phase Error Compensation for Near-Field Source Localization

In this subsection, we focus on estimating the locations of near-field radiating sources exploiting the array gain and phase error matrix Γ obtained by Algorithm 3. Specifically, we consider a scenario where four near-fieldsources are located at $\left(1250\;m,-35^{\circ }\right),\left(1450\;m,15.5^{\circ }\right),\left(1650\;m,20.1^{\circ }\right),\left(1840\;m,-5^{\circ }\right)$, respectively. In particular, for the following simulations, we consider the theoretical and estimated values of the gain and phase errors in Table 1. In addition, we search the range and azimuth of interest with steps 1m and $0.1^{\circ }$, respectively.

**Figure 6:**
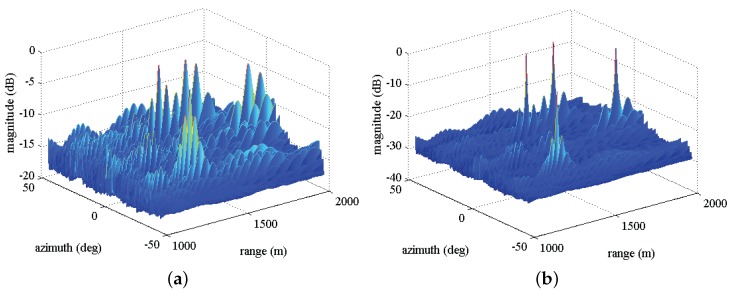
Two-dimensional MVDR spatial spectrums for four sources with the same SNR = 20 dB, (**a**) before calibration; (**b**) after calibration.

**Figure 7:**
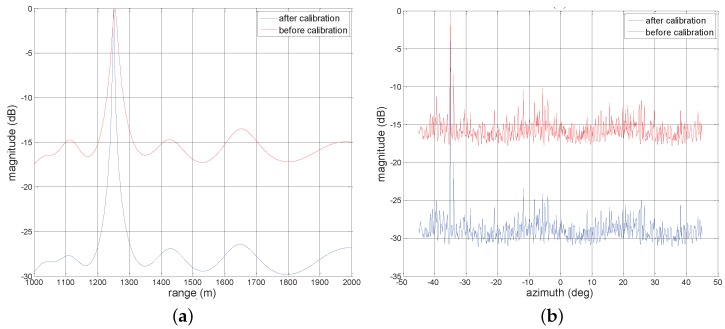
One-dimensional MVDR spatial spectrums for a source with estimated location $\left(1251\;m,-35^{\circ }\right)$ under SNR = 20 dB, (**a**) $\theta =-35^{\circ }$; (**b**) r=1251 m.

In Figure 6, we plot the two-dimensional MVDR spatial spectrums for four sources with the same SNR = 20 dB considering two cases of before compensation and after compensation. As expected, in Figure 6a, many high sidelobe levels emerge around the true targets locations due to the effect of array gain and phase errors. However, after exploiting Γ to compensate the nominal steering vector, in Figure 6b, we can observe that the spectrum peaks can be readily found, even though the estimation may be precise. Figure 7a,b depict one-dimensional MVDR spatial spectrums at $\theta =-35^{\circ }$ and r=1251 m for a source with the estimated location $\left(1251\;m,-35^{\circ }\right)$, respectively, considering two cases of before compensation and after compensation. The lower sidelobe levels are obtained compared with before compensation. For example, in Figure 7a, the peak sidelobe level (PSL) is about −13 dB before compensation, whereas it becomes about −27 dB after compensation.

**Figure 8:**
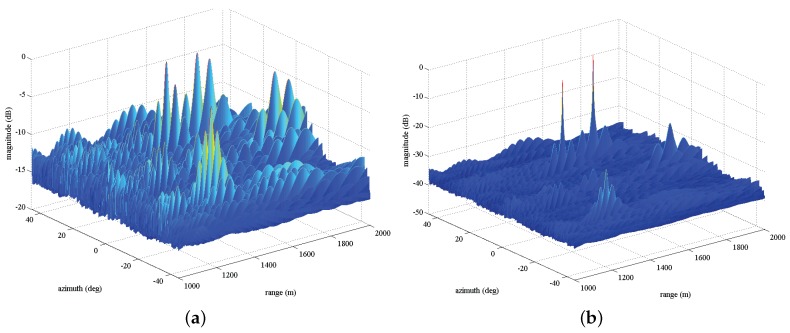
Two-dimensional MVDR spatial spectrums for four sources with different SNRs, (**a**) before calibration; (**b**) after calibration.

**Figure 9:**
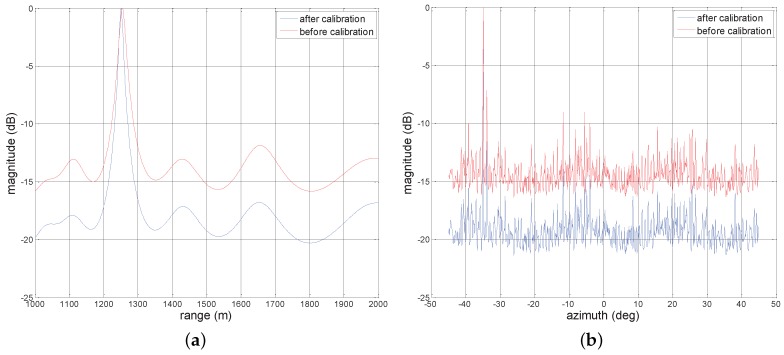
One-dimensional MVDR spatial spectrums for a source with estimated location $\left(1251\;m,-35^{\circ }\right)$ under SNR = 10 dB, (**a**) $\theta =-35^{\circ }$; (**b**) r=1251 m.

Next, we assess the estimation results of four sources $\left(1250\;m,-35^{\circ }\right),\left(1450\;m,15.5^{\circ }\right)$,$\left(1650\;m,20.1^{\circ }\right)$,

$\left(1840\;m,-5^{\circ }\right)$ with SNRs= 10 dB, 25 dB, 30 dB, 5 dB, respectively. In Figure 8, we provide the two-dimensional MVDR spatial spectrums for two cases of before compensation (Figure 8a) and after compensation (Figure 8b). We can observe that the peak of the weak target location (i.e., $\left(1840\;m,-5^{\circ }\right)$) drowns by high

sidelobe levels. In particular, the four peaks are more easy to obtain after compensation compared with Figure 8a. Furthermore, we show the one-dimensional MVDR spatial spectrums at $-35^{\circ }$ and 1251 m in Figure 9a,b, respectively, considering two cases of before compensation and after compensation. Particularly, the low sidelobe levels can be observed after compensation while showing higher sidelobe levels in comparison with Figure 7b since the low power. Finally, it is worth pointing out that these performance behaviors reflect that error parameters estimated by the devised algorithm can compensate manifold very well and significantly decrease the sidelobe level of MVDR spectrum.

In Table 2, we summarize the estimated values of the locations for four sources. Results exhibit that all estimated ranges share the error values of 1 m with the true values and there is no error for the obtained azimuths. Consequently, these performance behaviors again show the effectiveness of the proposed algorithm.

## 5. Conclusions

In this paper, we have addressed the near-field source location problem for a non-ULA embedded in gain and phase errors. We have presented a sequential optimization calibration algorithm to simultaneously estimate error parameters and locations of calibration sources under minimum variance estimation criterion exploiting some imprecise information. The source locations and the gain and phase errors are obtained iteratively with known ranges or azimuths at each iteration. Numerical results have shown that the array gain and phase errors obtained by the devised algorithm can compensate manifold very well and reduce MVDR spectrum sidelobe levels significantly. It is worth pointing out that the calibration method framework can be extended to other DOA algorithms, i.e., MUSIC, for arbitrary array geometries. Additionally, future work would be possible to consider the study of the convergence rate of the proposed sequential optimization algorithm. Last but not least, since the considered problem represents a case of optimization under uncertainty, it would be interesting to evaluate the integration of the proposed method with robust optimization and stochastic programming techniques [35,36,37].

## Figures and Tables

**Table 1:** Theoretical and average estimated values of the gain and phase errors for 12 sensors.

Sensor		1	2	3	4	5	6	7	8	9	10	11	12
Gain error	ρ	1.000	0.892	1.129	1.012	1.151	1.171	1.175	0.982	1.155	0.908	1.141	1.145
	$\hat{\rho }$	1.000	0.878	1.115	0.999	1.134	1.155	1.161	0.966	1.136	0.894	1.122	1.128
Gain error	φ(deg)	0	−4.067	−12.385	1.270	8.214	17.042	−1.542	11.344	14.826	−4.152	4.769	−4.553
	$\hat{\varphi }(\deg)$	0	−4.093	−12.249	1.453	8.285	17.232	−1.312	11.868	15.387	−3.443	5.580	−3.674

**Table 2:** Estimated values of the locations for four sources.

Target	1	2	3	4
Range (m)	1651	1451	1251	1841
Azimuth (deg)	20.1	15.5	−35	−5

## References

[1] Chen J.C., Yao K., Hudson R.E. (2002). Source localization and beamforming. IEEE Signal Process. Mag..

[2] Parr A., Miesen R., Vossiek M. (2016). Comparison of phase-based 3D near-field source localization techniques for UHF RFID. Sensors.

[3] Tiete J., Dominguez F., Silva B.D., Segers L., Steenhaut K., Touhafi A. (2014). SoundCompass: A distributed MEMS microphone array-based sensor for sound source localization. Sensors.

[4] Cobos M., Perez-Solano J.J., Felici-Castell S., Segura J., Navarro J.M. (2014). Cumulative-Sum-Based Localization of Sound Events in Low-Cost Wireless Acoustic Sensor Networks. IEEE/ACM Trans. Audio Speech Lang. Process..

[5] Canclini A., Antonacci E., Sarti A., Tubaro S. (2013). Acoustic source localization with distributed asynchronous microphone networks. IEEE Trans. Audio Speech Lang. Process..

[6] Meng W., Xiao W. (2017). Energy-Based Acoustic Source Localization Methods: A Survey. Sensors.

[7] Krim H., Viber M. (1996). Two Decades of Array Signal Processing Research: The Parametric Approach. IEEE Signal Process. Mag..

[8] Schmidt R. (1986). Multiple emitter location and signal parameter estimation. IEEE Trans. Aerosp. Electron. Syst..

[9] Capon J. (1969). High-resolution frequency-wavenumber spectrum analysis. Proc. IEEE.

[10] Cobos M., Marti A., Lopez J.J. (2011). A Modified SRP-PHAT Functional for Robust Real-Time Sound Source Localization With Scalable Spatial Sampling. IEEE Signal Proc. Lett..

[11] DiBiase J.H. (2000). A High Accuracy, Low-Latency Technique for Talker Localization in Reverberant Environments using Microphone Arrays. Ph.D. Thesis.

[12] Silverman H.F., Yu Y., Sachar J.M., Patterson W.R. (2005). Performance of real-time source-location estimators for a large-aperture microphone array. IEEE Trans. Speech Audio Process..

[13] Paulraj A., Kailath T. Direction of arrival estimation by eigenstructure methods with unknown sensor gain and phase. Proceedings of the IEEE International Conference on Acoustics, Speech, and Signal Processing.

[14] Wang M., Ma X., Yan S., Hao C. (2016). An Autocalibration Algorithm for Uniform Circular Array With Unknown Mutual Coupling. IEEE Antennas Wirel. Propag. Lett..

[15] Krishnappa G. (1981). Cross-spectral method of measuring acoustic intensity by correcting phase and gain mismatch errors by microphone calibration. J. Acoust. Soc. Am..

[16] Friedlander B., Weiss A. (1988). Eigenstructure methods for direction finding with sensor gain and phase uncertainties. Proc. Int. Conf. Acoust. Speech Signal Process..

[17] Weiss A.J., Friedlander B. (1990). Eigenstructure methods for direction finding with sensor gain and phase uncertainties. J. Circuits Syst. Signal Process..

[18] Ng B.P., Lie J.P., Er M.H., Feng A. (2009). A practical simple geometry and gain/phase calibration technique for antenna array processing. IEEE Trans. Signal Process..

[19] Viberg M., Swindlehurst A.L. (1994). A Bayesian approach to auto-calibration for parametric array signal processing. IEEE Trans. Signal Process..

[20] Liu A., Liao G., Zeng C., Yang Z., Xu Q. (2011). An eigenstructure method for estimating DOA and sensor gain-phase errors. IEEE Trans. Signal Process..

[21] Jiang J., Duan F., Chen J., Chao Z., Chang Z., Hua X. (2013). Two new estimation algorithms for sensor gain and phase errors based on different data models. IEEE Sens. J..

[22] Liao B., Chan S. (2013). Direction-of-arrival estimation in subarrays-based linear sparse arrays with gain/phase uncertainties. IEEE Trans. Aerosp. Electron. Syst..

[23] Cao S., Ye Z., Xu D., Xu X. (2013). A Hadamard product based method for DOA estimation and gain-phase error calibration. IEEE Trans. Aerosp. Electron. Syst..

[24] Liao B., Chan S. (2015). Direction finding in partly calibrated uniform linear arrays with unknown gains and phases. IEEE Trans. Aerosp. Electron. Syst..

[25] Liang J., Liu D., Zeng X., Wang W., Zhang J., Chen H. (2011). Joint azimuth-elevation/(-range) estimation of mixed near-field and far-field sources using two-stage separated steering vector-based algorithm. Prog. Electromagn. Res..

[26] Zaman F., Qureshi I.M., Naveed A., Khan Z.U. (2012). Joint estimation of amplitude, direction of arrival and range of near field sources using memetic computing. Prog. Electromagn. Res..

[27] Huang Y., Barkat M. (1991). Near-field multiple source localization by passive sensor array. IEEE Trans. Antennas Propag..

[28] Challa R.N., Shamsunder S. High-order subspace-based algorithms for passive localization of near-field sources. Proceedings of the 1995 Conference Record of the Twenty-Ninth Asilomar Conference on Signals, Systems and Computers.

[29] Yuen N., Friedlander B. (1998). Performance analysis of higher order ESPRIT for localization of near-field sources. IEEE Trans. Signal Process..

[30] Wu Y., So H., Hou C., Li J. (2007). Passive localization of near-field sources with a polarization sensitive array. IEEE Trans. Antennas Propag..

[31] Jiang J., Duan F., Chen J., Li Y., Hua X. (2013). Mixed near-field and far-field sources localization using the uniform linear sensor array. IEEE Sens. J..

[32] Xie D., Huang J., Ge H. Localization of near-field sources with partly calibrated subarray-based array. Proceedings of the 2010 the 5th IEEE Conference on Industrial Electronics and Applications.

[33] Yuan L., Jiang R., Chen Y. (2014). Gain and phase autocalibration of large uniform rectangular arrays for underwater 3-D sonar imaging systems. IEEE J. Ocean. Eng..

[34] Wax M., Kailath T. (1985). Detection of Signals by Information Theoretic Criteria. IEEE Trans. Acoust. Speech Signal Process..

[35] Bauschert T., Busing C., D’Andreagiovanni F., Koster A.C.A., Kutschka M., Steglich U. (2014). Network planning under demand uncertainty with robust optimization. IEEE Commun. Mag..

[36] D’Andreagiovanni F., Nardin A. (2015). Towards the fast and robust optimal design of Wireless Body Area Networks. Appl. Soft Comput..

[37] Shapiro A., Dentcheva D., Ruszczyński A. (2009). Ruszczynski: Lectures on Stochastic Programming: Modeling and Theory.

